# Combined physical and biological contributions to radiotherapy enhancement by Lu-based nanoscintillators in pancreatic cancer models

**DOI:** 10.7150/ntno.115120

**Published:** 2025-06-23

**Authors:** Sarah Stelse-Masson, Xenie Lytvynenko, Kristel Bedregal-Portugal, Clémentine Aubrun, Matéo Lavaud, Malika Kadri, Thibault Jacquet, Christine Moriscot, Benoit Gallet, Benoit Chovelon, Jean-Luc Coll, Jean-Luc Ravanat, Eva Mihóková, Václav Čuba, Hélène Elleaume, Anne-Laure Bulin

**Affiliations:** 1 Grenoble Alpes University, INSERM U1209, CNRS UMR5309, Institute for Advanced Biosciences, Cancer Targets and Experimental Therapeutics team, 38000 Grenoble, France.; 2 Grenoble Alpes University, INSERM UA7, Synchrotron Radiation for Biomedicine, 38000 Grenoble, France.; 3 Faculty of Nuclear Sciences and Physical Engineering, Czech Technical University in Prague, Prague 1, 115 19, Czech Republic.; 4 Grenoble Alpes University, EMBL, Integrated Structural Biology Grenoble (ISBG), UAR3518 CNRS CEA, 38000 Grenoble, France.; 5 Grenoble Alpes University, Institute of Structural Biology, CNRS CEA, 38000 Grenoble, France.; 6 Grenoble Alpes University, Département de Pharmacochimie Moléculaire, CNRS UMR 5063, 38000 Grenoble, France.; 7 Service de Biochimie SB2TE, Institut de Biologie et Pathologie, CHU Grenoble Alpes, 38041 Grenoble Cedex, France.; 8 Grenoble Alpes University, CEA, CNRS, Grenoble INP, SyMMES UMR 5819, 38000 Grenoble, France.; 9 Institute of Physics, Czech Academy of Sciences, Cukrovarnická 10, Prague, 162 00, Czech Republic.

**Keywords:** nanoscintillators, radiotherapy, radiation dose-enhancement, radiosensitization, pancreatic cancer

## Abstract

***Rationale:*** Pancreatic cancer has a dismal prognosis and requires better treatments. One promising approach aims at improving radiotherapy using nanoscintillators, which down-convert ionizing radiation into visible light, triggering various radiotherapeutic effects upon X-ray irradiation. One such effect is radiation dose-enhancement, driven by high-Z elements present in the nanoscintillator core. These elements efficiently absorb X-rays, releasing secondary electrons that amplify the radiation dose in the surrounding tissue.

***Methods:*** In this paper, we study the ability of Lu_3_Al_5_O_12_:Pr@SiO_2_, a lutetium-based nanoscintillator, to exert a radiation dose-enhancement effect in two human pancreatic cancer cell models, namely PANC-1 and MIA PaCa-2.

***Results:*** Lu_3_Al_5_O_12_:Pr@SiO_2_ nanoparticles showed negligible toxicity up to 1 mg/mL in 2D and 3D models. Using monochromatic synchrotron radiation, we demonstrated that a subtoxic nanoparticle concentration enhances the radiation dose in 3D spheroids in an energy-dependent manner. These results were further supported by Monte Carlo simulations. Beyond this physical contribution, γ-H2AX foci quantification revealed a biological component to the radiosensitization: Lu_3_Al_5_O_12_:Pr@SiO_2_ nanoparticles not only amplified initial DNA damage, but also impaired its repair.

***Conclusion:*** These findings highlight the dual contribution of Lu_3_Al_5_O_12_:Pr@SiO_2_ nanoparticles to radiotherapy enhancement, combining both physical dose-enhancement and biological modulation of DNA repair.

## Introduction

Pancreatic ductal adenocarcinoma remains one of the deadliest cancers in the world, with an average five-year survival rate of only 7%. It is currently the third leading cause of cancer death in the United States 1 and predicted to rise to become the second by the year 2030 2, highlighting the need for new strategies to manage the disease. Currently, surgery is the only curative treatment option, but less than 20% of patients are eligible for surgery, and incomplete resection remains a major issue 3. In order to reduce rates of recurrence and improve survival outcomes, adjuvant therapies such as chemotherapy and radiation therapy have been explored, with varying degrees of success. While adjuvant chemotherapy consistently improves survival outcomes and is included in the current standard of care 3,4, the benefit of radiotherapy remains less clear. Although it has demonstrated ability to improve local control, it has not led to improved survival outcomes for patients 4,5. Promising results are emerging with dose-escalated radiotherapy approaches 5,6, but these strategies pose the risk of increasing toxicity to adjacent healthy tissue. Thus, there is a clear interest in developing radiotherapeutic approaches to pancreatic cancer with increased specificity towards tumor tissue. Photodynamic therapy (PDT) is a light-based cancer therapy that has shown promising preclinical and clinical results for the treatment of pancreatic cancer 7,8. Although it may be better suited as a combination therapy, it shows promise as an adjuvant or local treatment to maximize therapeutic outcomes 8. PDT relies on the activation of photosensitive molecules by visible light, resulting in the generation of cytotoxic reactive oxygen species (ROS) that lead to tumor cell death via necrosis or apoptosis. While this therapy has several advantages, such as minimal toxicity to healthy tissue, lack of resistance mechanisms, and the ability to induce an inflammatory reaction linked to the development of systemic immunity 9,10, its application is limited primarily by the shallow penetration depth of light in tissues 9,11,12.

Several strategies are being explored to extend PDT to deeper-seated tumors, one of which relies on the use of X-rays as the activating source in a process called X-ray induced PDT (X-PDT). This process can be mediated by scintillating nanoparticles, or nanoscintillators, which down-convert ionizing radiation into lower energy photons in the visible range 13,14. When conjugated to or located nearby photosensitizers, scintillating nanoparticles can act as local light sources excited by X-rays to induce X-PDT, overcoming the penetration depth issue of activation by visible light 14,15. In this study, we selected Lu_3_Al_5_O_12_:Pr^3+^ as a scintillating material. This material, which has been extensively studied when in crystal shape, is well known for its strong radioluminescence emission peaking around 400 nm 16-19 that overlaps well with the absorption of many porphyrin-based photosensitizers. Previous work has shown the successful synthesis of Lu_3_Al_5_O_12_:Pr^3+^@SiO_2_ and demonstrated that such nanoparticles are capable of activating the photosensitizer Protoporphyrin IX (PpIX) to generate singlet oxygen under X-ray irradiation 20, thus showing promise as an X-PDT agent.

In addition to the possibility of inducing X-PDT *via* conjugation to photosensitizers, scintillating nanoparticles can induce several other important anti-tumor effects, most notably the radiation dose-enhancement (RDE) effect induced by high atomic number (high-Z) elements. When irradiated with orthovoltage X-rays (<250 keV), high-Z materials absorb X-ray photons more efficiently than surrounding tissues, thus generating ionization events, leading to the production of photoelectrons, Auger, and Compton electrons that increase local energy deposition 21,22. This concept was first described in the early 1980s by Callisen, Norman and collaborators, who used a molecular iodinated contrast agent to enhance the radiation dose 23,24. Later, Hainfeld *et al.* introduced the use of nanoparticles composed of high-Z elements to improve accumulation within tumor tissue, thereby opening the field of nanoparticle-enhanced radiotherapy 25,26. Subsequent studies demonstrated that sufficient intratumoral uptake of high-Z elements, followed by X-ray irradiation, significantly increases the delivered radiation dose and induces greater DNA damage compared to conventional radiotherapy alone 27. Several recent studies have demonstrated the ability of high-Z nanoscintillators to enhance tumor killing in preclinical models via this RDE effect 28-31. We expect Lu_3_Al_5_O_12_:Pr^3+^@SiO_2_ nanoparticles to also be capable of inducing a strong dose-enhancement effect. Before investigating the complex X-PDT effect of this compound in biological models, it is important to first more thoroughly understand the contribution of the RDE effect and the parameters that drive its efficacy. In this paper, we present a study of the ability of Lu_3_Al_5_O_12_:Pr^3+^@SiO_2_ nanoscintillators to induce an RDE effect in two pancreatic cancer cell lines, PANC-1 and MIA PaCa-2, grown in monolayers or as 3D spheroid models.

## Methods

### Synthesis of Lu_3_Al_5_O_12_:Pr^3+^@SiO_2_ nanoscintillators

Lu_3_Al_5_O_12_:Pr^3+^ (1%) nanopowder was prepared using a photo-induced precipitation method 32. All chemical reagents, including nitric acid, ammonium hydroxide (25-29%, p.a., PENTA) and absolute ethanol (≥ 99.8%, p.a., PENTA) were used without further purification and purchased from commercial sources. Prior to synthesis, Pr^3+^ stock solution was prepared by dissolving of PrO_2_-x (≥ 99.999%, Koch-Light Laboratories) in concentrated nitric acid. Reaction solution containing 3x10^-3^ mol/L of lutetium nitrate pentahydrate (99.999%, Sigma-Aldrich), 5x10^-3^ mol/L of aluminum nitrate nonahydrate (99.997%, Sigma-Aldrich), 0.1 mol/L of ammonium formate (≥ 99.995%, Sigma-Aldrich) and 3x10^-5^ mol/L of was prepared in deionized water. The solution was irradiated for 3.5 h with 4×25W low-pressure mercury lamps. After irradiation the obtained gelatinous product was filtered from the solution by microfiltration using 0.45 μm HAWP membrane filter (Millipore Ltd.) and dried in air. Lu_3_Al_5_O_12_:Pr^3+^ nanopowder was obtained by calcination of the milled precursor at 1100 °C for 2 h in a 0415 VAK vacuum furnace (Clasic). 200 mg of the Lu_3_Al_5_O_12_:Pr^3+^ nanopowder was dispersed in 40 mL of absolute ethanol in ultrasonic bath and then placed on a magnetic stirrer. 23 μL of tetraethoxysilane (TEOS; ≥99%, Sigma-Aldrich) was added to the suspension. After that, 7 mL of ammonium hydroxide solution was added dropwise to the mixture and vigorously stirred for 12 hours. The obtained silica-coated nanoparticles were washed 3x with water and dried in air.

### Characterization of Lu_3_Al_5_O_12_:Pr^3+^@SiO_2_ nanoscintillators

X-ray diffraction (XRD) measurements were performed using Rigaku MiniFlex 600 diffractometer equipped with Cu X-ray tube (Kα 1,2 = 0.15418 nm) with high voltage and current settings 40 kV and 15 mA, respectively. The collected data were evaluated and compared to standards using ICDD PDF-2 database, version 2013. The crystallite size of the nanoscintillators was evaluated by applying the Halder-Wagner method 33 using FWHM (full width at the half maximum) and the Scherrer constant K = 0.94 for calculations.

Transmission electron microscopy (TEM) images of the powder samples were collected by the transmission electron microscope JEOL JEM-3010 9JEOL (Ltd., Tokyo, Japan) equipped with EDX detector (Oxford Instruments, Abingdon, UK); high voltage acceleration was available up to 300 kV.

Dynamic light scattering (DLS) was used for determining the particle size distribution. DLS measurements were performed at room temperature (RT) with Zetasizer - Ultra Red (Malvern Instruments, Malvern, UK). The measurements were done in a 10 mm glass cell with square aperture. The suspension samples were prepared by dispersion of the powder in distilled water medium in ultrasonic bath for 1 h; the sample's volume was approximately 1.5 mL.

Room temperature radioluminescence spectra were measured upon polychromatic X-ray irradiation delivered by the SARRP irradiator (220 kV, 13 mA, IRMaGe Platform, GIN Grenoble). The radioluminescence signal was collected using an optical fiber and entered a Kymera 328i spectrograph (Andor, Oxford Instruments). The light was diffracted using a 150 l/mm grating blazed at 300 nm and the radioluminescence spectra was acquired using a Newton EM-CCD camera (Andor) was measured using an EM-CCD (Newton, Andor, Oxford Instruments).

### Cell culture and reagents

Cell lines PANC-1 and MIA PaCa-2 were obtained from The American Type Culture Collection. PANC-1 and MIA PaCa-2 are human pancreatic cancer cell lines derived from primary pancreatic ductal adenocarcinomas in male patients. They were maintained in Dulbecco's Modified Eagle's Medium (DMEM, Gibco) supplemented with 10% fetal bovine serum (Dominique Dutscher) and 1% penicillin/streptomycin (Gibco). The cultures were typically passaged weekly and maintained at standard culture conditions (37 °C, 5% CO_2_) at a passage number below 30.

### 3D cultures

The tumor spheroids were grown on Matrigel (Corning) in 24-well plates (Falcon) as previously described 34. Briefly, 200 μL of Matrigel was deposited into each well of a 24 well-plate, which was kept on ice. The Matrigel was then allowed to solidify for 20 minutes at 37 °C. After Matrigel deposition, PANC-1 or MIA PaCa-2 cells were seeded at a density of 7500 cells/well in a volume of 1 mL culture medium. The spheroids were allowed to grow for 5 days at culture conditions (37 °C, 5% CO_2_) before the addition of nanoparticles. The growth medium was refreshed as needed, typically every 3-5 days.

### Preparation of Lu_3_Al_5_O_12_:Pr^3+^@SiO_2_ stock solution

The suspension of Lu_3_Al_5_O_12_:Pr^3+^@SiO_2_ nanoparticles was prepared before each experiment using the following protocol: Lu_3_Al_5_O_12_:Pr^3+^@SiO_2_ powder was resuspended in phosphate-buffered saline (PBS) at a concentration of 2 mg/mL and sonicated in a Bioruptor bath sonicator (Diagenode) for 15 minutes. Dilutions were then prepared from this stock solution. DLS size and zeta potential measurements were performed on this resuspension diluted to 0.1 mg/mL in PBS using a Zetasizer Nano ZS (Malvern Panalytical) with the following parameters: material refractive index = 1.83, dispersant refractive index = 1.34, viscosity = 1.1 cP. Measurements were performed on at least three samples prepared independently.

### Toxicity

#### Experiments performed in 2D

PANC-1 and MIA PaCa-2 cells were seeded into 96-well plates at a density of 7500 cells/well in a volume of 50 μL culture medium and left to grow for 24 hours at 37 °C. The Lu_3_Al_5_O_12_:Pr^3+^@SiO_2_ stock solution was then diluted in medium to reach 2X the desired nanoparticle concentrations. 50 μL of each diluted nanoparticle suspension was added to the appropriate wells, bringing the total volume of each well to 100 μL. After a 24-hour incubation, the nanoparticle suspensions were removed and the cells were rinsed with 1x PBS. The viability of the cultures was assessed using the CellTiter 96 Aqueous One Solution Cell Proliferation Assay (Promega). 100 μL of diluted CellTiter 96 Aqueous One Solution Reagent (20 μL reagent in 100 μL culture medium) was added to each well. After 2 hours of incubation, the absorbance of the plate was read at 490 nm using a CLARIOstar Plus 96 well plate reader (BMG Labtech). Data was obtained from 3 wells per condition, and the experiment was performed 3 times per cell line.

#### Experiments performed in 3D models

Suspensions of Lu_3_Al_5_O_12_:Pr^3+^@SiO_2_ nanoparticles were prepared at the desired concentrations by diluting the stock solution in complete medium. The medium was removed from each well and replaced with 0.5 mL of the appropriate nanoparticle suspension. The cultures were returned to the incubator for 24 hours, after which the nanoparticle suspension was removed and each well was rinsed with 1x PBS. The live/dead staining protocol was applied to assess the viability of the cultures. Data was obtained from at least 50 spheroids per condition, and the experiment was performed twice per cell line.

### Treatment effect

#### Clonogenic assay to assess the treatment effect in 2D

PANC-1 or MIA PaCa-2 cells were seeded into T25 cell culture flasks (Falcon) at a density of 50 000 cells/flask. After 4 days of growth, the medium was removed from each flask and replaced by 2 mL of Lu_3_Al_5_O_12_:Pr^3+^@SiO_2_ (0.5 mg/mL) suspension in culture medium. After 24 hours of incubation at 37 °C, the nanoparticle suspension was removed, the cells were rinsed once with 1x PBS, and 5 mL of fresh medium was added to each flask. The flasks were irradiated at 2 or 4 Gy (CIX2 irradiator, CEA). After irradiation, the cells from each flask were collected using trypsin (trypsin-EDTA 0.5%, Gibco) and counted. Dilutions were prepared to seed them into 6-well plates (Falcon) according to the densities found in Table 1. Colonies were left to grow at 37 °C for two weeks, with the culture medium being refreshed as needed. After the colonies had sufficiently grown (typically two weeks) the cells were washed with 1x PBS and stained with a solution of 0.5% crystal violet (Amresco) and 6.0% glutaraldehyde (Sigma Aldrich). The number of colonies in each well was counted using ImageJ, and the survival fraction for each condition was calculated. Data was obtained from 6 wells per condition (including two different seeding densities), and the experiment was performed at least twice per cell line. Data was fitted to a linear quadratic model using Prism. For each cell line, the control survival curve (without nanoparticles) was fitted using the linear quadratic mode, providing the alpha and beta parameters that are characteristic of the cell line under these irradiation conditions (Figure 3). The survival curve obtained in the presence of nanoparticles was then fitted using the alpha and beta parameters obtained with the control data and the dose multiplied by the DEF factor. The fit of the survival data provided the DEF factors, as described in more detail in 31. Significance was calculated using a two-way ANOVA followed by Tukey post hoc test, where (*) indicates p < 0.05, (**) indicates p < 0.01, (***) indicates p < 0.001, and (****) indicates p < 0.0001.

#### Viability assay to study treatment efficacy in 3D

After 5 days of growth, the medium was removed from the wells and replaced with a suspension of Lu_3_Al_5_O_12_:Pr^3+^@SiO_2_ nanoparticles (0.5 mg/mL) in culture medium. After 24 hours of incubation at 37 °C, the nanoparticle suspension was removed, the wells were rinsed once with 1x PBS, and 1 mL of fresh medium was added to each well. The cultures were irradiated at 2, 4, or 8 Gy (62.3 or 64.3 keV) using a monochromatic beam (ID17 biomedical beamline at European Synchrotron Radiation Facility, ESRF, Grenoble, France). After irradiation, the cultures were returned to the incubator for 6 days, then the live/dead staining protocol was applied to assess the viability of the cultures. Data was obtained from at least 100 spheroids per condition, and the experiment was performed once. Significance was calculated using a two-way ANOVA followed by Tukey post hoc test, where (*) indicates p < 0.05, (**) indicates p < 0.01, (***) indicates p < 0.001, and (****) indicates p < 0.0001.

#### Live/dead protocol to study the viability of 3D cultures

A control of completely necrotic cells (total killing control) was prepared according to the following protocol: spheroids were fixed with 10% formalin (Sigma Aldrich) for 2 minutes at room temperature, then rinsed once with 1x PBS and permeabilized with a 0.5% Triton-X 100 (Bio-Rad) solution for 30 minutes at room temperature. Spheroids were then rinsed twice with 0.1 M glycine (Euromedex) and maintained in 1x PBS. To assess viability, the culture medium (or PBS in the case of the total killing group) was removed from each well and replaced with 0.5 mL of a staining solution containing 2 μM calcein AM (Invitrogen) and 3 μM propidium iodide (PI) (Sigma Aldrich) in PBS. After 1 hour of incubation at 37 °C, the cultures were imaged using confocal laser scanning microscopy (LSM510 ConfoCor II Combination system, Zeiss) through a 5x objective (Plan Neofluar, NA = 0.15). The fluorescence signals were recorded at λ_exc_ = 488 nm / λ_em_ = 500-540 nm (calcein) and λ_exc_ = 543 nm / λ_em_ = 600-670 nm (PI). Two images were taken per well. Image analysis was performed according to CALYPSO methodology as described previously 34.

### Uptake - ICP-MS

#### Analysis of 2D grown cells

PANC-1 and MIA PaCa-2 cells were seeded into 6-well plates at a density of 50 000 cells/well in a volume of 2 mL culture medium. After 4 days of growth, the medium from each well was removed and replaced by 1 mL Lu_3_Al_5_O_12_:Pr^3+^@SiO_2_ (0.5 mg/mL) suspension in culture medium. After 24 hours of incubation at 37 °C, the nanoparticle suspension was removed and each well was rinsed with 1x PBS. The cells were then trypsinized and washed with PBS, then resuspended in 100 μL PBS for analysis. A PierceTM BCA protein quantification assay (ThermoFisher) was performed for each sample. The quantity of lutetium (^175^Lu isotope) contained in each sample was determined by quadrupole ICP-MS (Perkin Elmer NexION 2000, Waltham, MA, USA). Samples were mineralized under atmospheric pressure in nitric acid for 24 h at room temperature, followed by three phases of 8 h in an oven (50 °C) over 3 consecutive days. The mineralization was diluted to reach 1% concentration of nitric acid before analysis. Standard solutions were prepared in nitric acid 1% v/v. ^103^Rh was used as an internal standard. The experiment was performed on three samples collected independently. Significance was calculated using an unpaired t-test, with (*) indicating p < 0.05.

#### Analysis of 3D grown cells

After 5 days of growth, the medium was removed from the wells and replaced with a suspension of Lu_3_Al_5_O_12_:Pr^3+^@SiO_2_ (0.5 mg/mL) in culture medium. After 24 hours of incubation at 37 °C, the nanoparticle suspension was removed and each well was rinsed with 1x PBS. 1 mL of cell recovery solution (Corning) was added to each well to dissolve the Matrigel. The contents of each well were transferred to a 15 mL tube (Falcon) and kept on ice for 2 hours. When spheroids were seen accumulating at the bottom of the tubes, the tubes were centrifuged (5 min, 1200 rpm) and washed twice with cold PBS. The samples were resuspended in several hundred microliters and stored at -20 °C until ICP-MS analysis. A PierceTM BCA protein quantification assay (ThermoFisher) was performed for each sample. Quantification of lutetium was determined by ICP-MS as described in the previous section. Data was collected from 6 wells per condition, and the experiment was performed on two samples collected independently.

### Transmission electron microscopy

PANC-1 cells were grown on Lab-Tek^TM^ chamber slides (Thermo Scientific) for two days, after which medium was removed and replaced with a suspension of Lu_3_Al_5_O_12_:Pr^3+^@SiO_2_ nanoparticles (0.5 mg/mL) in culture medium. After 24 hours of incubation, the cells were washed with DMEM and fixed for 30 min in a solution containing 2% paraformaldehyde (PFA) (R1026) and 0.2% glutaraldehyde (GA, R1020) in DMEM with gentle shaking. After successive fixation, rinsing and staining steps as previously described 35, cells were dehydrated in graded ethanol series, and embedded in Epon resin (Embed 812, Electron Microscopy Sciences, 14120). Ultrathin sections of 70 nm were cut on an ultramicrotome (Leica, UC7), collected on formvar-carbon-coated copper 100 mesh grids (Formvar) and imaged with a Tecnai G2 Spirit BioTwin, with magnifications from 690x to 9300x.

### Immunofluorescence staining

12 mm glass coverslips (Knittel) were sterilized and placed in the wells of 24-well plates. PANC-1 and MIA PaCa-2 cells were seeded into the wells at a density of 30 000 cells/well in a volume of 1 mL culture medium. After 48 hours of growth, the medium was removed and replaced with 0.5 mL of Lu_3_Al_5_O_12_:Pr^3+^@SiO_2_ (0.5 mg/mL) suspension in culture medium. After 24 hours of incubation at 37 °C, the nanoparticle suspension was removed, the cells were rinsed once with 1x PBS, and 1 mL of fresh medium was added to each well. The cultures were irradiated at 2 Gy (SARRP irradiator, GIN) and returned to the incubator. After 1 hour or 24 hours, the wells were rinsed 2x with Tris-buffered saline (TBS), then fixed with 2% PFA (prepared from 10% formalin, diluted in TBS) for 10 minutes at 4 °C. The wells were rinsed 3x with TBS and stored at 4 °C until the next day. The cells were then permeabilized with 0.2% Triton-X 100 (Bio-Rad) in TBS for 10 minutes at room temperature, rinsed once with TBS, and blocked in 5% bovine serum albumin (BSA) (Interchim) in TBS for 1 hour. After an additional TBS rinse, the coverslips were then incubated with primary antibody for 2 hours at room temperature. The coverslips were then washed 3 times again with TBS and incubated with secondary antibody for 1 hour in the dark at room temperature. After 3 more TBS washes, the coverslips were stained with 2 μM Hoechst for 5 minutes at room temperature, rinsed once more with TBS, and mounted onto glass slides using fluorescence mounting media (Agilent). After 30 minutes of polymerization at room temperature and storage at 4 °C, the slides were imaged at 63X (Water immersion, NA=1.2) using a Stellaris 8 confocal microscope (Leica Microsystems). The nuclei were imaged upon excitation at λ_exc_ = 405 nm (diode) and light emission was collected between 415 and 509 nm. γ-H2AX foci were imaged upon an excitation delivered by the white laser at λ_exc_ = 499 nm; emission was collected between 509 and 639 nm. Z-stacks of approximately 6 μm were acquired with a step of 0.358 μm. Primary antibody: anti-Phospho-Histone H2AX Ser139 clone 20E3 (Cell Signaling, 1:200 in 1% BSA in TBS. Secondary antibody: goat anti-rabbit Alexa Fluor 488 (Invitrogen, 1:500 in 1% BSA in TBS). Foci were quantified using an ImageJ macro (Bram van den Broek, The Netherlands Cancer Institute). The images displayed here were processed using ImageJ in the following way to remove background signal: the nuclei and foci channels were separated and background subtraction was performed on the foci channel. The nuclei channel was then thresholded and applied as a mask to the foci channel to remove any signal not localized to the nuclei. Data was collected from at least 80 nuclei per condition, and the experiment was performed once. Significance was calculated using a two-way ANOVA followed by Tukey post hoc test, where (*) indicates p < 0.05, (**) indicates p < 0.01, (***) indicates p < 0.001, and (****) indicates p < 0.0001.

### Irradiation conditions

#### Polychromatic irradiation source - CEA

X-rays were delivered by a CIX2 irradiator (XStrahl) through a 3 mm aluminum filter, with the following parameters: voltage = 195 kV, current = 10 mA, and focal source distance = 40 cm. The cell culture plates were placed at the center of the irradiation field to ensure a uniform radiation dose. The dose rate was measured using a PTW ionization chamber (TN30010-1) and a PTW UNIDOS E electrometer. The typical dose rate in water for these experiments was 1.8 ± 0.5 Gy/min.

#### Polychromatic irradiation source - GIN

X-rays were delivered by a SARRP irradiator (XStrahl, SAXO - Grenoble IRMaGe facility) through a 0.15 mm copper filter, with the following parameters: voltage = 220 kVp, current = 13 mA, focal source distance = 35 cm. Samples were positioned within the homogeneous area of the irradiation field. The typical dose rate in culture medium for these experiments was 3.1 ± 1.0 Gy/min.

#### Monochromatic irradiation source - ESRF

Radiotherapy was delivered by monochromatic X-ray beams on the medical beamline (ID17) at ESRF (beam time number: md1260). The ESRF unit was operated in 7/8 mode. The w125 wiggler was set at 50 mm, and the beam filters were 0.8 mm carbon and 3 mm aluminum.

Cell cultures were irradiated in 24-well plates, either 1 keV below, or 1 keV above, the lutetium K-edge (63.31 keV, energy bandwidth of ~ 70 eV). The plate containing the cells was tilted at 26° to the incident X-ray beam and scanned vertically through the X-ray beam (45 mm wide and 1 mm high) at a speed of 2.5 mm/s to irradiate a total height of 65 mm. The number of scans required to deliver the prescribed dose was calculated on the basis of the ring current (mA) and the X-ray dose rate measured using an ionization chamber (UNIDOS PTW 31 002, Freiburg, Germany) read by a PTW electrometer (PTW UNIDOS E, Freiburg, Germany). The typical dose rate in water for the experiment was 3.9 ± 0.1 mGy/s/mA.

### Monte Carlo simulations

Geant4 (version 4.10.1, patch 01) was used to simulate the spectra of secondary particles (photons and electrons) generated during the primary interaction between the incoming X-rays and Lu_3_Al_5_O_12_:Pr^3+^. The Livermore low energy package was used as a Physics List, as previously described 30,36. Briefly, a rod of Lu_3_Al_5_O_12_:Pr^3+^ (1 nm^2^ area and 1 mm long) was created and placed in vacuum. The Lu_3_Al_5_O_12_:Pr^3+^ rod was virtually exposed to an incoming flux of 1x10^6^ monochromatic photons (at energies of 63.31 keV and 64.31 keV, that is, 1 keV above and below the Lu K-edge). These photons were hitting the rod at the center of its 1 nm^2^ surface. All secondary particles (electrons and photons) generated during this irradiation were collected and quantified by a virtual detector placed symmetrically around the Lu_3_Al_5_O_12_:Pr^3+^ rod.

## Results

### Lu_3_Al_5_O_12_:Pr^3+^@SiO_2_ nanoscintillators were successfully synthesized using a photo-induced precipitation method

**Figure 1:**
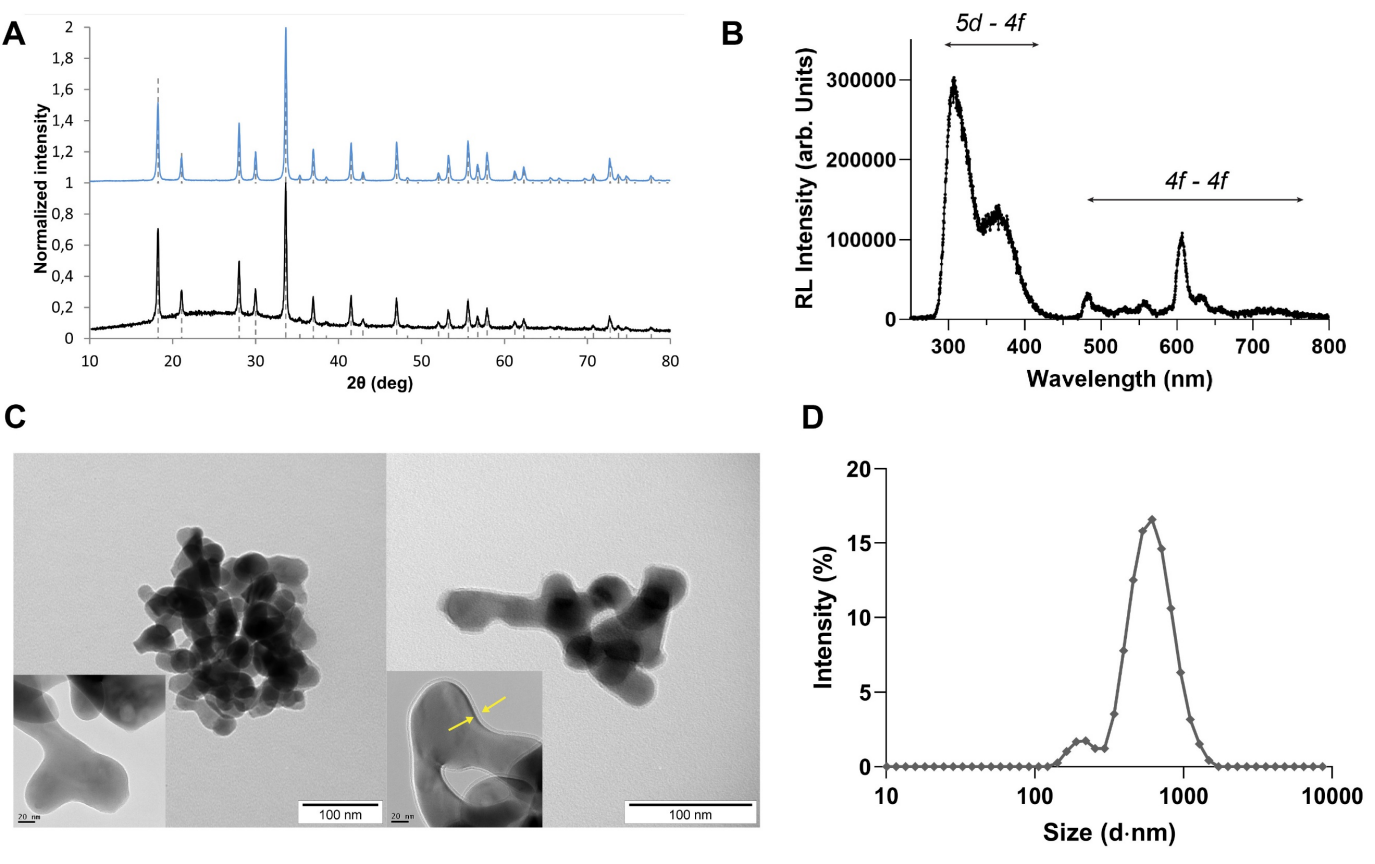
**Lu_3_Al_5_O_12_:Pr^3+^ and Lu_3_Al_5_O_12_:Pr^3+^@SiO_2_ nanoscintillators are well crystallized and exhibit a strong radioluminescence signal. A)** Diffractograms obtained by X-ray Diffraction Spectroscopy of Lu_3_Al_5_O_12_:Pr^3+^ (top) and Lu_3_Al_5_O_12_:Pr^3+^@SiO_2_ (bottom) samples compared with standard lines from ICDD PDF-2 database (dashed lines, card No. 01-073-1368). **B)** Room temperature radioluminescence spectrum of the Lu_3_Al_5_O_12_:Pr^3+^@SiO_2_ nanopowder shows the typical emission of Pr^3+^: the broad emission corresponding to the 5d-4f transition and the narrow peaks corresponding to the 4f-4f transitions. **C)** TEM images of non-modified Lu_3_Al_5_O_12_:Pr^3+^ (left) and silica-coated Lu_3_Al_5_O_12_:Pr^3+^@SiO_2_ (right). Scale = 100 nm. In the inset, the detail of SiO_2_ coating is shown (indicated by yellow arrows); Scale = 20 nm. **D)** Particle size distribution of lyophilized Lu_3_Al_5_O_12_:Pr^3+^@SiO_2_ nanoparticles after resuspension in phosphate-buffered saline (PBS), measured by Dynamic Light Scattering (DLS). Measurements were performed on at least three samples prepared independently.

The nanoparticles were synthesized using a photo-induced precipitation method, and subsequently coated with a SiO_2_ layer. This coating plays several roles; it provides means for surface passivation and helps limit particle aggregation. In addition, the silica shell provides a versatile platform for future conjugation and functionalization through surface reactions. X-ray diffractograms performed on the prepared Lu_3_Al_5_O_12_:Pr^3+^ and Lu_3_Al_5_O_12_:Pr^3+^@SiO_2_ nanoparticles after calcination are shown in Figure 1.A. The diffraction patterns are consistent with the standard parameters of Lu_3_Al_5_O_12_:Pr^3+^ from the ICDD PDF-2 database (card No. 01-073-1368) and correspond to the cubic crystal structure and Ia
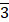
d space group. As shown by the narrow peaks, the solid phase is well-crystallized and the crystallite size of Lu_3_Al_5_O_12_:Pr^3+^ and Lu_3_Al_5_O_12_:Pr^3+^@SiO_2_ was determined as (44 ± 6) nm and (46 ± 6) nm, respectively. A slight background intensity increase observed in the range 17-37° 2θ (Figure 1.B) confirms the amorphous character of the silica layer.

Room temperature radioluminescence spectrum (Figure 1.B) shows the typical emission bands of Pr^3+^ centers related to 4f - 4f transitions (480-650 nm) and broad emission bands peaking at 315 nm and 370 nm due to 5d - 4f transitions.

TEM imaging performed on nanoparticles before and after SiO_2_ modification show that the nanoparticles present a rounded shape and a slight grain intergrowth. In addition, nanoparticles appear to agglomerate, which is due to the high temperature annealing step, necessary to obtain a crystal phase. The silica encapsulation process leads to the formation of a relatively smooth SiO_2_ layer on the nanoparticle surface with a measured thickness of about 2.5 nm, which is in good agreement with the results obtained from XRD measurements. When dispersed in distilled water, particle size distribution obtained by DLS method results in a mean particle size of (99 ± 6) nm and (106 ± 6) nm for Lu_3_Al_5_O_12_:Pr^3+^ and Lu_3_Al_5_O_12_:Pr^3+^@SiO_2_ samples, respectively. DLS measurements performed in PBS after the resuspension protocol yielded a higher particle size of 617 ± 46 nm and a polydispersity index (PDI) of 0.41 ± 0.05, indicating that Lu_3_Al_5_O_12_:Pr^3+^@SiO_2_ nanoparticles tend to aggregate upon resuspension. The zetasizer was also used to measure the zeta potential of the particle suspension in PBS, which was determined to be -27.4 ± 0.7 mV. To maintain consistent size distribution across experiments, a fresh Lu_3_Al_5_O_12_:Pr^3+^@SiO_2_ suspension was prepared before each experiment using the same batch of lyophilized nanopowder, followed by sonication before use. DLS measurements were also performed in culture medium (DMEM 10% FBS), yielding a particle size of 489 nm ± 136 nm and a PDI of 0.57 ± 0.05, reaching the same order of magnitude of what was observed in PBS.

### Lu_3_Al_5_O_12_:Pr^3+^@SiO_2_ nanoparticles demonstrate limited toxicity and uptake in 2D cultures of pancreatic cancer cells

Our first objective was to characterize the uptake and toxicity of Lu_3_Al_5_O_12_:Pr^3+^@SiO_2_ nanoparticles in 2D cultures of pancreatic cancer cells. We first used Transmission Electron Microscopy (TEM) to visualize the internalization of the nanoparticles into PANC-1 cells.

**Figure 2:**
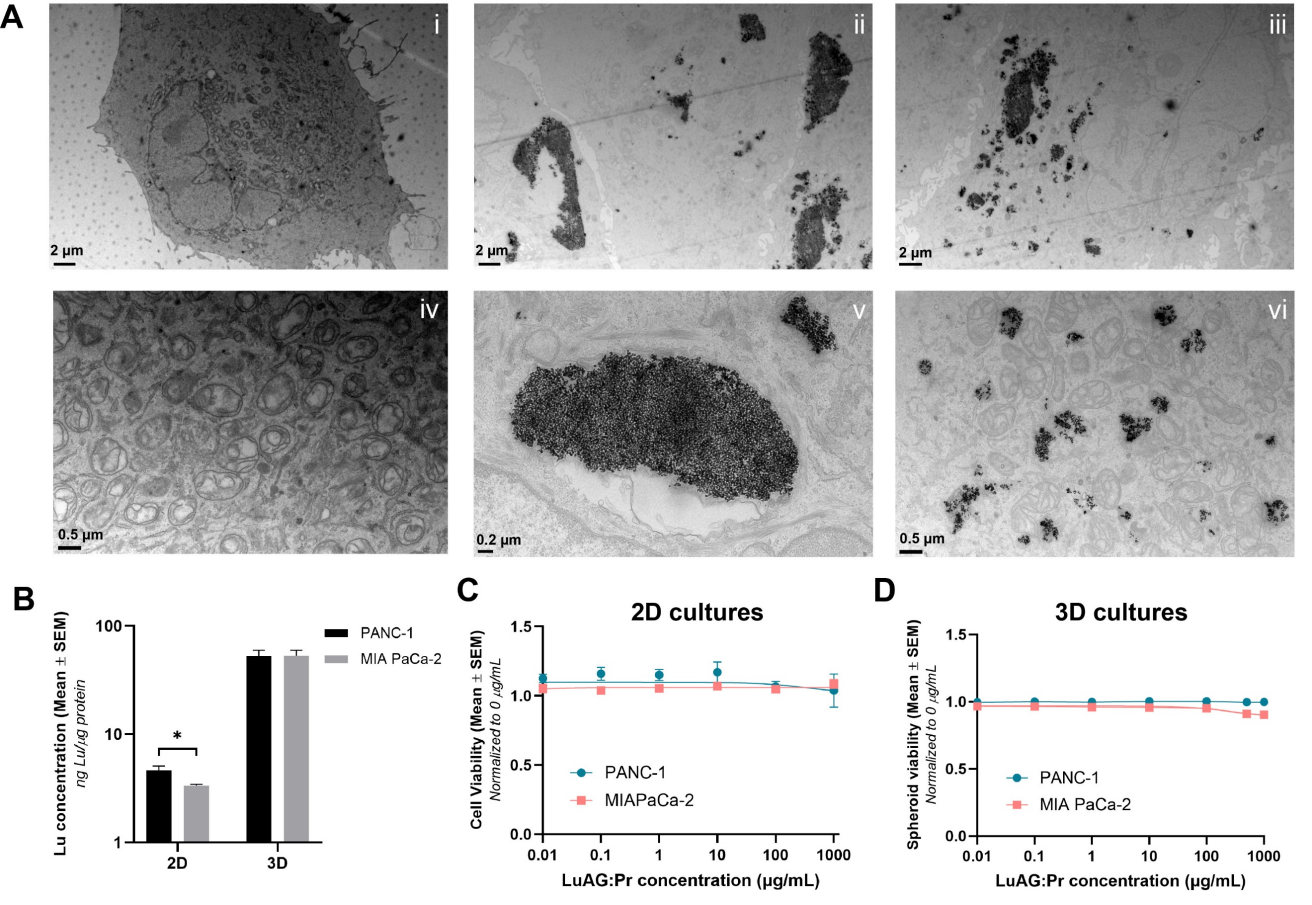
** Lu_3_Al_5_O_12_:Pr^3+^@SiO_2_ nanoparticles show limited uptake and low toxicity in 2D and 3D cultures of pancreatic cancer cells. A)** Representative TEM images of control PANC-1 cells (i, iv) and PANC-1 cells previously incubated with 0.5 mg/mL Lu_3_Al_5_O_12_:Pr^3+^@SiO_2_ suspension for 24 hours (ii, iii, v, vi). Scale= 2 μm (i-iii), 0.5 μm (iv, vi) or 0.2 μm (v). **B)** Quantity of Lutetium detected (ng) per μg of protein in 2D and 3D PANC-1 and MIA PaCa-2 cell cultures after 24-hour incubation with 0.5 mg/mL Lu_3_Al_5_O_12_:Pr^3+^@SiO_2_ suspension. Data were normalized to protein content measured using a Pierce BCA protein assay and are presented as mean ± SEM. Results were pooled from at least two independent replicates, and significance was calculated using an unpaired t-test, with (*) indicating p < 0.05. **C)** Cell viability determined by MTS assay (mean ± SEM) and **D)** spheroid viability determined by live/dead assay (mean ± SEM) plotted as a function of Lu_3_Al_5_O_12_:Pr^3+^@SiO_2_ concentration after a 24-hour incubation, normalized to a no nano control. Results were obtained from 3 wells (2D experiments) or 50 spheroids (3D experiments) per condition, and pooled from at least two independent experiments.

**Figure 3:**
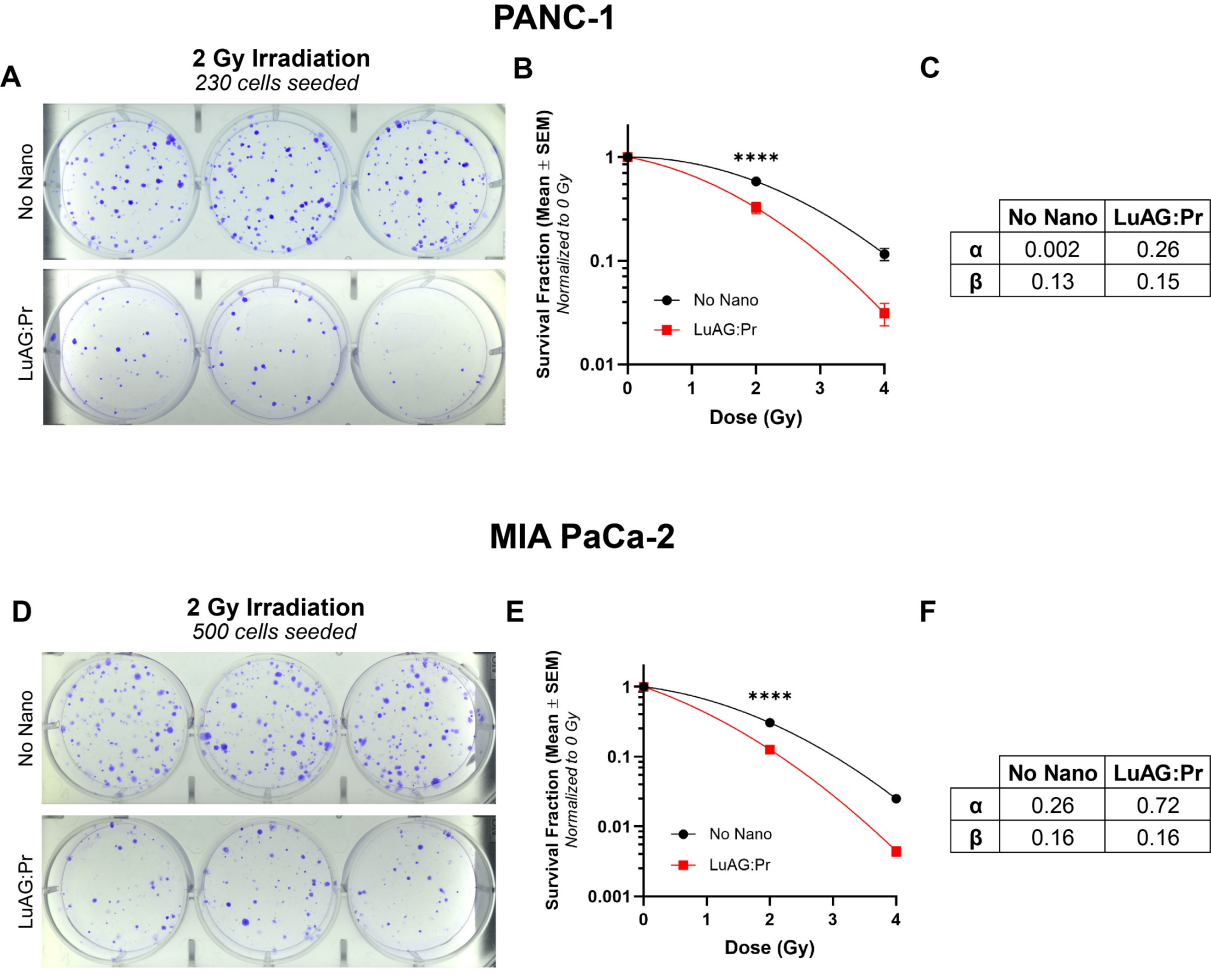
** Lu_3_Al_5_O_12_:Pr^3+^@SiO_2_ (LuAG:Pr) nanoparticles reduce proliferation of pancreatic cancer cells under X-ray irradiation**. Representative images of plates seeded with treated **A)** PANC-1 cells and **D)** MIA PaCa-2 cells, at a density of 230 and 500 cells, respectively. Cells were irradiated with 2 Gy after 24 hours of incubation with 0 (control, top panels) or 0.5 mg/mL (bottom panels) Lu_3_Al_5_O_12_:Pr^3+^@SiO_2_ nanoscintillators. Survival fraction (mean ± SEM) plotted as function of radiation dose are shown for **B)** PANC-1 cells and **E)** MIA PaCa-2 cells. The survival fractions were normalized to the 0 Gy condition for each treatment group. Results were collected from 6 wells per condition from at least 2 independent experiments. Data was fitted to a linear quadratic model using Prism to determine α and β values for **C)** PANC-1 and **F)** MIA PaCa-2 cells, and significance was calculated using a two-way analysis of variance (ANOVA) followed by Tukey post hoc test. (*) indicates p < 0.05, (**) indicates p < 0.01, (***) indicates p < 0.001, and (****) indicates p < 0.0001.

Images i and iv of Figure 2.A show control PANC-1 cells (with no nanoparticles) imaged at two different magnifications. The top image shows the entirety of a PANC-1 cell: the borders of the cell membrane can be seen, as well as the nucleus and other intercellular structures. The image below, taken at higher magnification, shows the mitochondria of the cell in more detail. Images ii, iii, v, and vi show PANC-1 cells after a 24-hour incubation with 0.5 mg/mL of Lu_3_Al_5_O_12_:Pr^3+^@SiO_2_ suspension. In the top images, which were taken at a lower magnification, nanoparticles are seen to have been internalized in the form of aggregates. These aggregates appear in a range of sizes up to the order of micrometers, confirming that Lu_3_Al_5_O_12_:Pr^3+^@SiO_2_ nanoparticles present a high particle size and polydispersity upon resuspension. Images v and vi, taken at higher magnification, show some of these larger and smaller aggregates in more detail. Overall, the uptake of Lu_3_Al_5_O_12_:Pr^3+^@SiO_2_ into PANC-1 cells was observed to be quite inhomogeneous, with some cells internalizing very large aggregates, others internalizing smaller aggregates, and some taking up no nanoparticles at all.

While TEM imaging provides qualitative information about the intracellular localization of nanoparticles, it does not allow us to quantify the amount of Lu_3_Al_5_O_12_:Pr^3+^@SiO_2_ taken up by the cells. ICP-MS, on the other hand, can provide a quantitative measure of the amount of lutetium actually accumulated in cells. In addition to internalization in 2D, we are also interested in how much lutetium accumulates in 3D spheroid models of pancreatic cancer cells. According to the ICP-MS results shown in Figure 2.B, 2D PANC-1 cultures showed slightly higher levels of lutetium per μg of protein compared to MIA PaCa-2 cultures, indicating higher internalization of Lu_3_Al_5_O_12_:Pr^3+^@SiO_2_ in PANC-1 compared to MIA PaCa-2 cells. However, in 3D cultures, the quantity of lutetium per μg of protein was determined to be the same in both cell lines. Additionally, more than 10 times the amount of lutetium was detected per μg of protein in 3D cultures than in 2D cultures. This could indicate that much of the nanoparticles taken up into the spheroids were not actually internalized by the cells but remained trapped extracellularly within the spheroids.

Cell viability data determined by an MTS assay (Figure 2.C) indicate that Lu_3_Al_5_O_12_:Pr^3+^@SiO_2_ nanoparticles do not exhibit noticeable toxicity against PANC-1 or MIA PaCa-2 cells up to a concentration of 1 mg/mL in culture medium after a 24-hour incubation. Similarly, the nanoparticles did not induce toxicity upon incubation with 3D PANC-1 and MIA PaCa-2 cultures up to a concentration of 1 mg/mL as determined by the live/dead assay (Figure 2.D).

### Lu_3_Al_5_O_12_:Pr^3+^@SiO_2_ nanoscintillators reduce the proliferation of PANC-1 and MIA PaCa-2 cells under X-ray irradiation

We evaluated the efficacy of Lu_3_Al_5_O_12_:Pr^3+^@SiO_2_ nanoparticles under X-ray irradiation in 2D cell cultures, using a clonogenic assay to assess the impact on the cells' ability to proliferate after treatment. X-ray irradiation was delivered using a polychromatic source, as detailed in the *Methods* section. The results of the clonogenic assay showed that a 24-hour incubation with 0.5 mg/mL Lu_3_Al_5_O_12_:Pr^3+^@SiO_2_ is able to significantly reduce the survival fraction of both PANC-1 and MIA PaCa-2 cells under 2 Gy and 4 Gy of X-ray irradiation, compared to radiation alone. As shown by lower cell survival after irradiation, MIA PaCa-2 are more radiosensitive compared to PANC-1 (Figure S1), which is consistent with previous reports 37. By fitting the data to a linear quadratic model, we obtained the curves' α and β values, which represent two different contributions to cell killing. While the linear term α represents the contribution from "single-hit" events, the quadratic term β reflects the contribution from "multiple-hit" cell death resulting from the combination of sub-lethal events 38. Using these results, we were able to calculate the radiation dose-enhancement factor (DEF) provided by Lu_3_Al_5_O_12_:Pr^3+^@SiO_2_ nanoscintillators, which was determined to be 1.4 for both PANC-1 and MIA PaCa-2 cultures under these incubation conditions.

### Lu_3_Al_5_O_12_:Pr^3+^@SiO_2_ nanoscintillators increase the DNA damage production during radiotherapy and impair the DNA repair mechanisms

**Figure 4:**
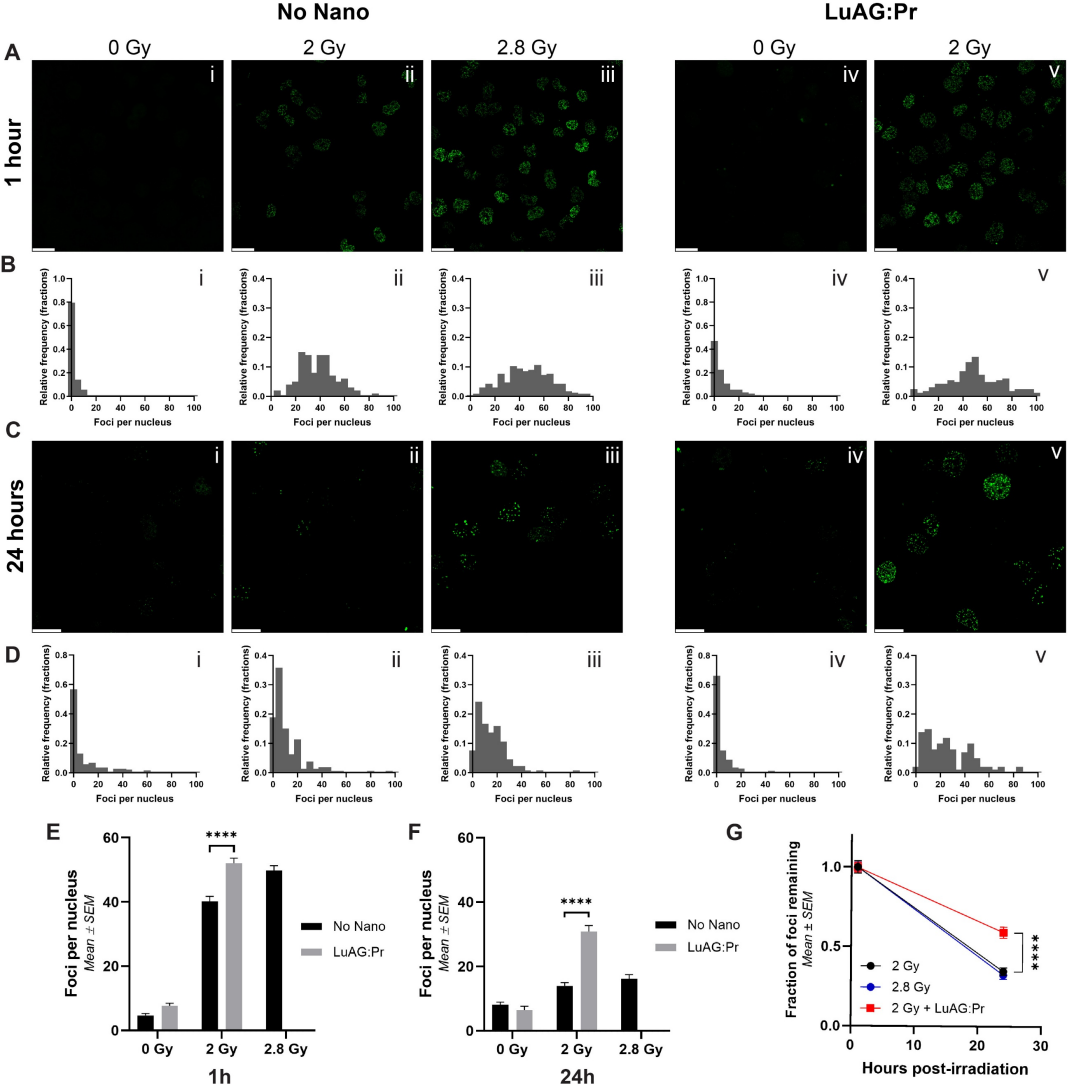
** Lu_3_Al_5_O_12_:Pr^3+^@SiO_2_ (LuAG:Pr) nanoparticles significantly enhance the number of DNA double-stranded breaks in PANC-1 cells immediately after X-ray irradiation and 24 hours after X-ray irradiation. A)** and **C)** show representative microscopy images taken of PANC-1 cells collected either 1 hour or 24 hours post-irradiation, respectively. In images i-iii, immunofluorescence staining for γ-H2AX foci was performed on PANC-1 cells after they received 0, 2, or 2.8 Gy of X-rays. In images iv-v, cells were first incubated with 0.5 mg/mL Lu_3_Al_5_O_12_:Pr^3+^@SiO_2_ for 24 hours, then received either 0 or 2 Gy of X-rays. Scale bar = 25 μm. The number of foci per nucleus was quantified, and the frequency distributions for each condition are shown in panels **B)** and **D)** for cells collected 1 hour and 24 hours after irradiation, respectively. The average number of foci per nucleus (mean ± SEM) in cells irradiated with 2 Gy of X-rays is shown in graphs **E)** and **F)** for samples collected 1 hour or 24 hours post-irradiation, respectively. At both timepoints, the number of foci in cells incubated with Lu_3_Al_5_O_12_:Pr^3+^@SiO_2_ was significantly higher than those that received irradiation alone. **G)** presents the fraction of foci remaining at 24 hours post-irradiation normalized to the number of foci present at 1 hour post-irradiation for the 2 Gy, 2.8 Gy, and 2 Gy + Lu_3_Al_5_O_12_:Pr^3+^@SiO_2_ conditions. Foci quantification data was taken from at least 80 nuclei per condition. Statistical significance was assessed using a two-way ANOVA followed by Tukey post hoc test. (*) indicates p < 0.05, (**) indicates p < 0.01, (***) indicates p < 0.001, and (****) indicates p < 0.0001.

In order to understand if the reduction in clonogenicity was correlated with an increase in DNA double-stranded break (DSBs), we used immunofluorescence staining to quantify the amount of γ-H2AX foci present in PANC-1 cells 1 hour after X-ray irradiation, when signal from γ-H2AX is expected to be at a near-maximum (Figure S2). We also quantified the amount of foci remaining in cell nuclei 24 hours after irradiation (Figure S3), as it has been shown that the number of foci remaining after 24 hours corresponds to the fraction of cells that fail to proliferate 39. In Figure 4.A, images i, ii, and iii show nuclei of PANC-1 cells that received 0, 2, or 2.8 Gy of X-rays, respectively, in the absence of any nanoparticles. 2.8 Gy was chosen as it is the X-ray dose that corresponds to the effective dose delivered due to the RDE effect induced by Lu_3_Al_5_O_12_:Pr^3+^@SiO_2_ under 2 Gy of X-rays (=2 Gy*DEF = 2 Gy*1.4). Images iv and v show nuclei of PANC-1 cells that received 0 or 2 Gy of X-rays after a 24-hour incubation with 0.5 mg/mL of Lu_3_Al_5_O_12_:Pr^3+^@SiO_2_ nanoparticles. The respective frequency distributions of the number of foci per nucleus for each condition can be seen in Figure 4.B. We first observe that the signal from γ-H2AX appears to be higher in cells that received 2 Gy of X-rays after incubation with the nanoparticles than in cells that received 2 Gy of X-rays alone, and is more similar in intensity to the sample that received 2.8 Gy of X-rays. In quantifying the foci, we find that the average number of foci per nucleus is significantly higher in the 2 Gy + nano condition (52 foci/nucleus) than in the 2 Gy alone condition (40 foci/nucleus, Figure 4.E), confirming that Lu_3_Al_5_O_12_:Pr^3+^@SiO_2_ is able to increase the amount of DNA DSBs induced under X-ray irradiation. When looking at the frequency distributions, we can observe that the distribution of foci in the 2 Gy + nano condition more closely resembles that of the 2.8 Gy condition, with the addition of Lu_3_Al_5_O_12_:Pr^3+^@SiO_2_ increasing the number of nuclei with more than 60 foci under 2 Gy of irradiation.

A similar trend is observed in the samples collected 24 hours post-irradiation (Figure 4.C and 4.D). At this timepoint, only limited signal remains in the 2 Gy alone condition, and we observe the strongest signal in the 2 Gy + nano condition. In quantifying the number of foci per nucleus, we confirm that the average number of foci per nucleus is significantly higher in the 2 Gy + nano condition (31 foci/nucleus) than in the 2 Gy alone condition (14 foci/nucleus, Figure 4.F), indicating that Lu_3_Al_5_O_12_:Pr^3+^@SiO_2_ nanoparticles are able to increase the number of lethal DNA lesions induced by X-ray irradiation. Interestingly, the number of foci remaining in the 2 Gy + nanoparticles condition appears to be even higher than the number remaining in the 2.8 Gy condition (31 foci/nucleus vs 16 foci/nucleus). This is especially apparent in the frequency distributions; in the 2.8 Gy condition, most nuclei contain fewer than 40 foci, while in the 2 Gy + nanoparticles condition, a much higher number of nuclei contain more than 40 foci. This indicates that the effect of Lu_3_Al_5_O_12_:Pr^3+^@SiO_2_ under X-ray irradiation may be more complex than a physical dose-enhancement, as the effects of 2 Gy + nanoparticles are more pronounced than those induced by the expected "equivalent" dose of 2.8 Gy. More specifically, the presence of Lu_3_Al_5_O_12_:Pr^3+^@SiO_2_ nanoparticles may interfere with the cells' ability to repair DNA damage induced by X-ray irradiation. Figure 4.G shows the fraction of foci remaining 24 hours post-irradiation compared to those detected 1 hour post-irradiation for the various irradiation conditions. For cells irradiated in the absence of nanoparticles, the fraction of foci remaining after 24 hours was found to be around 33%, regardless of whether cells were irradiated with 2 Gy or 2.8 Gy of X-rays. For cells that were irradiated with 2 Gy after incubation with Lu_3_Al_5_O_12_:Pr^3+^@SiO_2_, this fraction was found to be significantly higher, with nearly 60% of foci remaining, suggesting that the nanoparticles may enhance the effects of X-rays not only by increasing the effective radiation dose but also through additional mechanisms that may impair DNA damage repair. However, the slower repair kinetics observed following irradiation in the presence of Lu_3_Al_5_O_12_:Pr^3+^@SiO_2_ nanoparticles may also be explained by an increased formation of multiple local damage sites known to be less efficiently repaired than DSBs. Formation of such clustered lesions could be due to the photoelectrons generated upon X-ray interactions with heavy elements, and could explain the radiation dose-enhancement effect observed in the presence of the nanoparticles 40. The experiment was repeated in MIA PaCa-2 cells (Figure S4), where we observed a similar modification in DNA damage repair kinetics.

### Lu_3_Al_5_O_12_:Pr^3+^@SiO_2_ nanoparticles induce radiation dose-enhancement in an X-ray energy-dependent manner in 3D pancreatic cancer cell cultures

We studied the effects of the radiation dose-enhancement induced by Lu_3_Al_5_O_12_:Pr^3+^@SiO_2_ nanoparticles on pancreatic cancer spheroid size and viability. In contrast to the 2D cultures, the 3D cultures were irradiated using monochromatic synchrotron radiation delivered at the European Synchrotron Radiation Facility (ESRF, Grenoble, France).

**Figure 5:**
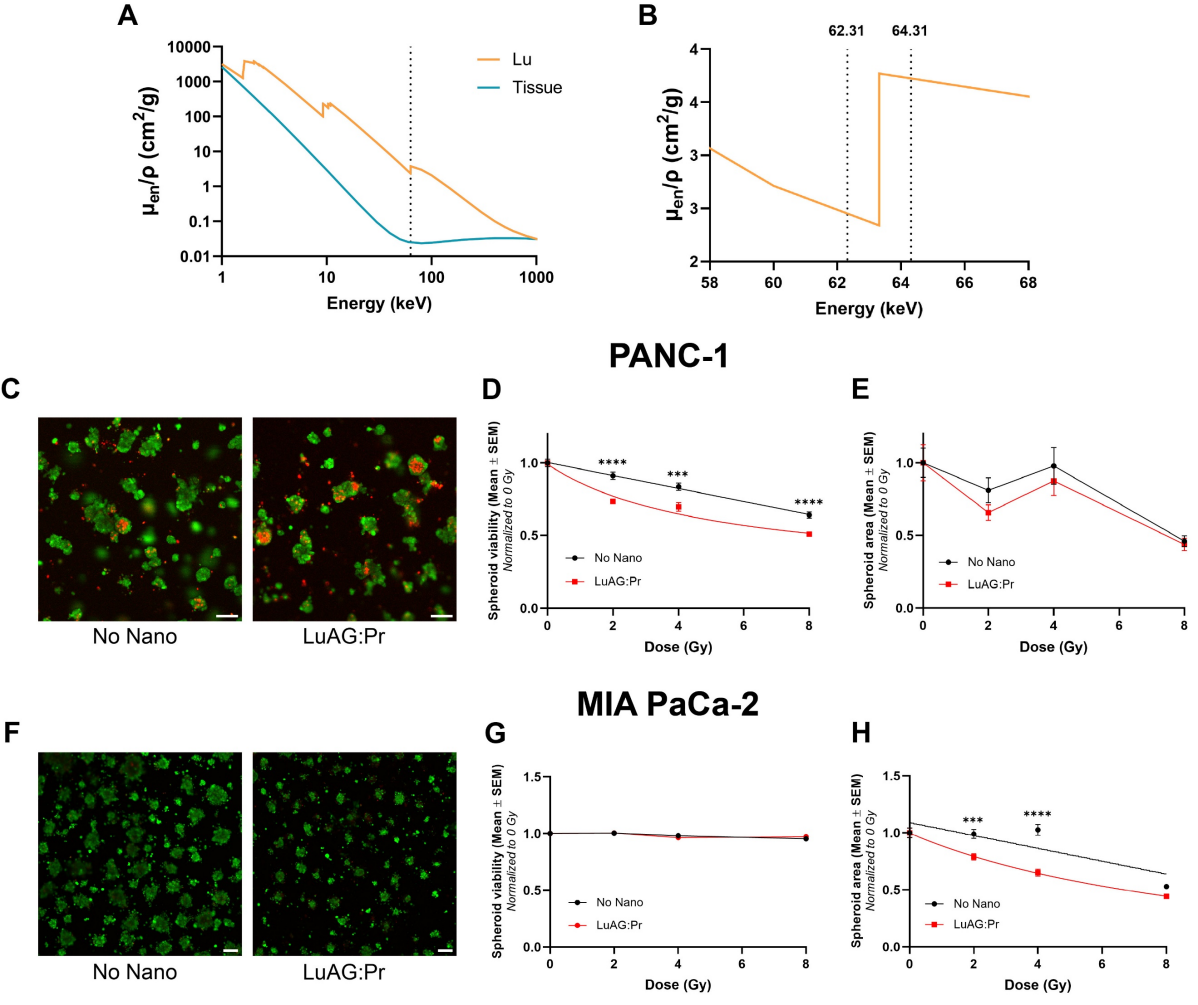
** Lu_3_Al_5_O_12_:Pr^3+^@SiO_2_ (LuAG:Pr) nanoparticles potentiate X-ray radiotherapy in 3D models of pancreatic tumors by impacting the viability or the spheroid area of PANC-1 and MIA PaCa-2 spheroids, respectively. A)** Mass energy absorption coefficients as a function of X-ray energy of lutetium (orange) and soft tissue (blue). Dashed line represents the Lu K-edge at 63.31 keV. Data obtained from 41. **B)** The K-edge of Lu (63.31 keV), as well as the irradiation energies used (62.31 keV, 64.31 keV, represented by dashed lines). **C)**, **F)** Representative live/dead images of PANC-1 (**C**) or MIA PaCa-2 (**F**) spheroids after 4 Gy of irradiation (64.31 keV) either without nanoparticles (left) or after a 24-hour incubation with 0.5 mg/mL Lu_3_Al_5_O_12_:Pr^3+^@SiO_2_ (right). Scale bar = 200 μm. Average PANC-1 spheroid viability (**D)** and area (**E**) (mean ± SEM) as a function of radiation dose determined by live/dead assay 6 days after irradiation above the Lu K-edge (64.31 keV). Average MIA PaCa-2 spheroid viability (**G)** and area (**H**) (mean ± SEM) as a function of radiation dose determined by live/dead assay 6 days after irradiation above the Lu K-edge (64.31 keV). Spheroid viability (D, G) or size (E, H) was normalized to the 0 Gy condition and fitted with a nonlinear regression in Prism according to the [inhibitor] versus response model with three parameters. Statistical significance was assessed using a two-way ANOVA followed by Tukey post hoc test. (*) indicates p < 0.05, (**) indicates p < 0.01, (***) indicates p < 0.001, and (****) indicates p < 0.0001.

We irradiated the cultures at 62.31 and 64.31 keV, which corresponds to 1 keV below and 1 keV above the lutetium K-edge, respectively, as illustrated in Figure 5.A and 5.B. Figure 5.A shows the mass-energy absorption coefficient of lutetium (orange) and soft tissue (blue) as a function of X-ray energy and Figure 5.B displays a magnified view of the K-edge of lutetium. Irradiating below and above the K-edge enables the investigation of the photoelectric effect, and thus, the physical origin of the radiation dose-enhancement effect. While cultures irradiated below the lutetium K-edge showed no sign of a radiation dose-enhancement effect after incubation with Lu_3_Al_5_O_12_:Pr^3+^@SiO_2_ nanoparticles (Figure S5.B), those irradiated above the K-edge did show enhanced response to X-ray irradiation upon incubation with Lu_3_Al_5_O_12_:Pr^3+^@SiO_2_. Interestingly, the effect of radiation damage on spheroid size and viability seemed to differ between the two cell lines. PANC-1 spheroids exhibited lower average viability when irradiated with Lu_3_Al_5_O_12_:Pr^3+^@SiO_2_ compared to irradiation without Lu_3_Al_5_O_12_:Pr^3+^@SiO_2_ (Figure 5.D); however, the average spheroid size remained unchanged (Figure 5.E). MIA PaCa-2 spheroids, on the other hand, did not show a change in viability in response to radiation dose-enhancement (Figure 5.G), but showed a strong reduction in size in response to the treatment (Figure 5.H).

### Monte Carlo simulations show that X-ray energy impacts the strength of the radiation dose-enhancement effect

Although Lu_3_Al_5_O_12_:Pr^3+^@SiO_2_ was seemingly able to produce a dose-enhancement effect in spheroids under 64.31 keV synchrotron radiation, the same effect was not observed at a lower X-ray energy of 62.31 keV (Figure S5). We hypothesize that this is due to the difference in X-ray absorption below and above the K-edge of lutetium (located at 63.31 keV), which could lead to higher production of photoelectrons and thus, a stronger radiation dose-enhancement effect. In order to investigate the strength of the effect at these two energies, we performed Monte Carlo simulations using Geant4 toolkit, to calculate the secondary particles generated in Lu_3_Al_5_O_12_:Pr^3+^ after interaction with monochromatic X-rays of 62.31 keV or 64.31 keV, respectively.

**Figure 6:**
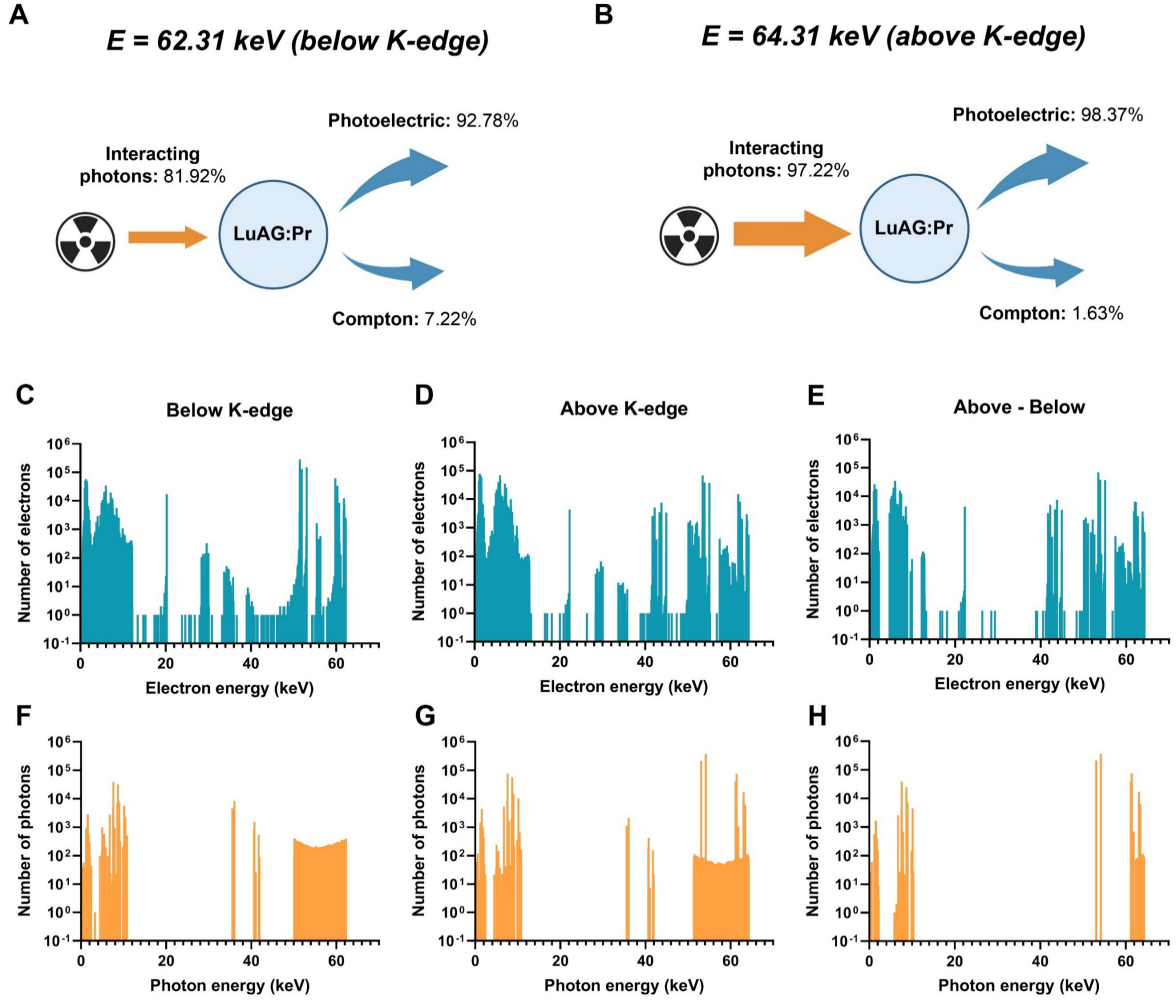
** Simulation of X-ray interactions with Lu_3_Al_5_O_12_:Pr^3+^ (LuAG:Pr) nanoparticles.** The percentage of interactions occurring through photoelectric or Compton effects are shown for **A)** 62.31 keV X-rays and **B)** 64.31 keV X-rays. The spectra of electrons and photons generated after interaction between an X-ray photon and Lu_3_Al_5_O_12_:Pr^3+^ for **C)**, **F)** 62.31 keV X-rays and **D), G)** 64.31 keV X-rays. **E)** and **H)** present the spectra of additional electrons (**E**) and photons (**H**) generated after the interaction with 64.31 keV X-rays compared to 62.31 keV X-rays.

The simulations confirmed several key findings: first, a higher percentage of incoming X-ray photons interact with Lu_3_Al_5_O_12_:Pr^3+^ when the X-ray energy is above the K-edge compared to when it is below the K-edge. Below the K-edge, only 81.92% of incoming photons interact with Lu_3_Al_5_O_12_:Pr^3+^; above the K-edge, this number rises to 97.22%. In addition to increasing the percentage of interacting photons, the higher X-ray energy also changes the distribution of processes that occur during interaction of photons with the material. While only 92.78% of interacting photons induce photoelectric effect below the K-edge, up to 98.37% of interacting photons induce photoelectric effect above the K-edge. Finally, the higher X-ray energy results in higher generation of secondary electrons and photons. This can be seen especially clearly in panels E and H of Figure 6, which represent the number of additional electrons and photons generated by 64.21 keV X-rays versus 62.21 keV X-rays upon interaction with Lu_3_Al_5_O_12_:Pr^3+^.

## Discussion

Among other factors, the strength of the radiation dose-enhancement effect largely depends on the local accumulation of nanoscintillators within tumor tissue 31. In this context, maximizing this effect requires nanoscintillator formulations to accumulate in the immediate vicinity of cancer cells and spheroids. While upon synthesis, Lu_3_Al_5_O_12_:Pr^3+^@SiO_2_ nanoparticles yield a particle size close to 100 nm 20, resuspension of powdered Lu_3_Al_5_O_12_:Pr^3+^@SiO_2_ in PBS prior to *in vitro* experiments often yielded a much higher particle size with high polydispersity, as shown in Figure 1.D, revealing the tendency of these particles to form large aggregates. These aggregates likely limited the uptake of the nanoparticles - indeed, TEM imaging revealed that the particles taken up by PANC-1 were mainly in the form of large aggregates on the order of microns. The internalization of smaller, more well-dispersed particles was also observed, but this tendency was not observed uniformly throughout the cells. ICP-MS results reveal a similar uptake of Lu_3_Al_5_O_12_:Pr^3+^@SiO_2_ by PANC-1 and MIA PaCa-2 cells, for both 2D and 3D cultures. However, in both cell lines, the amount of Lu_3_Al_5_O_12_:Pr^3+^@SiO_2_ normalized to protein concentration is much higher in 3D cultures than in 2D cultures. It is thus hypothesized that the Lu quantified in the 3D cultures was not actually internalized by the cells but remained trapped extracellularly within the spheroids. Despite limited internalization, Lu_3_Al_5_O_12_:Pr^3+^@SiO_2_ nanoparticles were able to induce a substantial radiation dose-enhancement effect resulting in reduced cell proliferation and increased DNA damage, suggesting that the species responsible for the radiation dose-enhancement effect may be able to reach their intracellular targets without efficient cellular internalization of Lu_3_Al_5_O_12_:Pr^3+^@SiO_2_. This points to an important contribution of the photoelectric effect, as photoelectrons generated upon X-ray interactions with heavy elements can travel distances on the order of a few tens of microns 14. The significant contribution of the photoelectric effect is also supported by the energy dependence we observed in the experiments performed upon monochromatic synchrotron radiation. The results of the Monte Carlo simulations showed that increasing the energy of X-rays above the K-edge of lutetium increases the contribution of the photoelectric effect, generating higher energy electrons that may be able to reach intracellular targets from further away, despite the nanoparticles not being internalized within the cells.

The radiation dose-enhancement effect is as a purely physical phenomenon, resulting from increased X-ray absorption by heavy elements, which leads to a higher effective dose delivered to the surrounding tissue. While this effect was originally demonstrated using gold nanoparticles, it has also been more recently demonstrated using nanoscintillators composed of heavy elements 30,31. During our investigation of the nanoscintillator Lu_3_Al_5_O_12_:Pr^3+^@SiO_2_, we verified the presence of such a physical effect by demonstrating the impact of X-ray energy on the strength of radiation dose-enhancement, a conclusion that was further supported by Monte Carlo simulations. However, changes in the DNA repair after Lu_3_Al_5_O_12_:Pr^3+^@SiO_2_-induced radiation dose-enhancement indicate that there is also a biological component involved. Incubation with Lu_3_Al_5_O_12_:Pr^3+^@SiO_2_ in the absence of X-ray irradiation does not induce any γ-H2AX foci, indicating no genotoxicity from the nanoparticles themselves; however, they are able to increase the amount of foci remaining 24 hours after irradiation with X-rays, indicating that they may interfere with DNA damage repair 45. The combination of physical dose-enhancement and biological modulation of DNA repair may lead to a more effective overall therapeutic response under X-ray irradiation.

This fundamental study demonstrates that radiotherapy efficacy can be improved though the interaction of physical and biological effects. However, two key aspects must be carefully considered before advancing toward clinical translation.

First, the relatively large size and aggregation tendency of the nanoparticles may hinder their systemic delivery due to rapid clearance by the reticuloendothelial system. Nonetheless, strategies to enhance colloidal stability are currently being pursued. In addition, intratumoral injection - a clinically relevant route of administration for dose-enhancing agents - is already being explored in clinical trials with HfO_2_ nanoparticles and could offer a viable strategy for achieving localized accumulation of larger nanoparticles prior to radiotherapy 42-44. While this approach may not be universally applicable, it presents a feasible and promising path for further development.

Second, the radiation dose-enhancement effects observed in this study were obtained using orthovoltage/low-energy X-rays, which differ from the high-energy (MeV) photons typically used in clinical external-beam radiotherapy. While this presents a potential limitation for direct clinical translation, understanding these effects at lower X-ray energies serves as a necessary first step in evaluating whether similar mechanisms could also contribute under clinically relevant irradiation settings. Moreover, while it is established that the efficacy of physical radiation dose-enhancement is highly dependent on X-ray energy, the energy dependence of the biological radiosensitization effect observed in our study remains unknown. This relationship requires further investigation. In particular, comparing the kinetics of DNA DSBs repair following exposure to low-versus high-energy X-rays would be a critical question to address in future studies. More generally, additional studies are needed to assess the behavior of these nanoparticles under megavoltage irradiation and to determine whether their effects can be extended to or optimized for high-energy clinical conditions. If the approach proves ineffective under clinically used megavoltage irradiation, an alternative clinical pathway could involve the use of brachytherapy. This technique employs internally placed radioactive sources that emit lower energy photons, potentially offering greater compatibility with the properties of the nanoscintillators investigated in this study.

## Conclusion

Our findings confirmed that Lu_3_Al_5_O_12_:Pr^3+^@SiO_2_ nanoscintillators, even when not functionalized with photosensitizers, are able to increase radiotherapy efficacy through a radiation dose-enhancement effect. As expected, we observed the contribution of a physical mechanism, as evidenced by the influence of the X-ray irradiation source. However, beyond this anticipated effect, we also observed a biological response, particularly in the modulation of DNA repair. This suggests that nanoscintillators not only physically enhance the radiation dose but may also interfere with cellular pathways responsible for DNA damage repair, increasing overall treatment efficacy.

We observed a physical dose-enhancement effect of Lu_3_Al_5_O_12_:Pr^3+^@SiO_2_ nanoparticles despite limited internalization into PANC-1 and MIA PaCa-2 cells. This finding, combined with our Monte Carlo simulations and synchrotron radiation experiments, highlight the important contribution of the photoelectric effect and suggest that nanoparticle internalization, while beneficial, may not be strictly necessary for achieving radiation dose-enhancement.

The observed biological effect, specifically the prolonged presence of γ-H2AX foci following irradiation in the presence of Lu_3_Al_5_O_12_:Pr^3+^@SiO_2_, indicates an impact on DNA repair kinetics. While the nanoparticles alone do not induce genotoxicity, they appear to modulate normal repair kinetics following X-ray irradiation, which could lead to a synergistic enhancement of radiotherapy efficacy. The contribution of both physical and biological mechanisms may explain the unexpectedly high therapeutic effect sometimes observed when studying high-Z nanoparticles under X-ray irradiation, even under conditions where a significant physical dose-enhancement is not expected.

Further studies will focus on understanding the specific pathways involved in this DNA repair modulation, in particular the potential role of nanoscintillators in altering repair protein recruitment and activity. A better understanding of how nanoscintillators interact with DNA repair processes will provide valuable insights for designing new nanoscintillators and optimizing treatment strategies that harness both physical and biological mechanisms of dose-enhancement.

## Supplementary Material

Supplementary figures.

## Figures and Tables

**Table 1:** Clonogenic assay seeding densities

	PANC-1	MIA PaCa-2
**0 Gy**	200, 300	200, 300
**2 Gy**	230, 345	300, 500
**4 Gy**	260, 390	2500, 5000
