# Supplementary material for: Combined physical and biological contributions to radiotherapy enhancement by Lu-based nanoscintillators in pancreatic cancer models
Source: Nanotheranostics. 2025 Jun 23;9(3):199–215. doi: 10.7150/ntno.115120 (PMC12435256; doi:10.7150/ntno.115120)
Supplement: Supplementary file 1 — Supplementary figures. [file ntnov09p0199s1.pdf]

Supplementary Information

Combined physical and biological contributions to radiotherapy enhancement by Lu-based nanoscintillators in pancreatic cancer models

Sarah Stelse-Masson, Xenie Lytvynenko, Kristel Bedregal-Portugal, Clémentine Aubrun, Matéo Lavaud, Malika Kadri, Thibault Jacquet, Christine Moriscot, Benoit Gallet, Benoit Chovelon, Jean-Luc Coll, Jean-Luc Ravanat, Eva Mihóková, Václav Čuba, Hélène Elleaume, Anne-Laure Bulin*

* Corresponding author, E-mail: anne-laure.bulin@inserm.fr

Intrinsic Radiosensitivity of PANC-1 vs MIA PaCa-2 cell lines

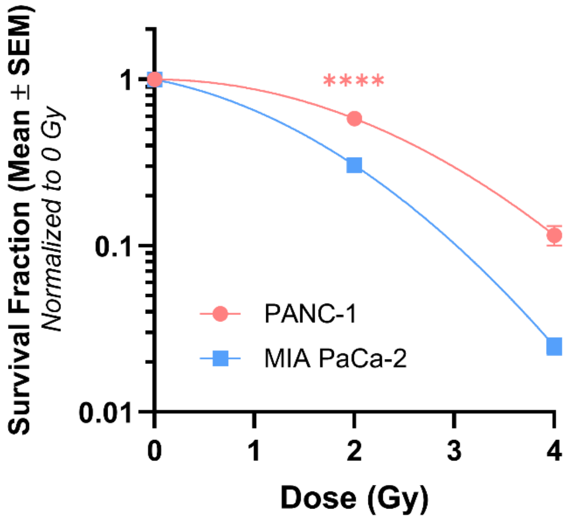

Figure S1: Survival fraction (mean ± SEM) of PANC-1 (pink) and MIA PaCa-2 (blue) cells plotted as a function of irradiation dose, normalized to the 0 Gy condition. Data was fitted to a linear quadratic model using Prism and significance was calculated using a two-way ANOVA. Results were collected from 6 wells per condition from at least 2 independent experiments. (*) indicates $p < 0.05$, (**) indicates $p < 0.01$, (***) indicates $p < 0.001$, and (****) indicates $p < 0.0001$.

γ-H2AX foci in PANC-1 cells after X-ray irradiation

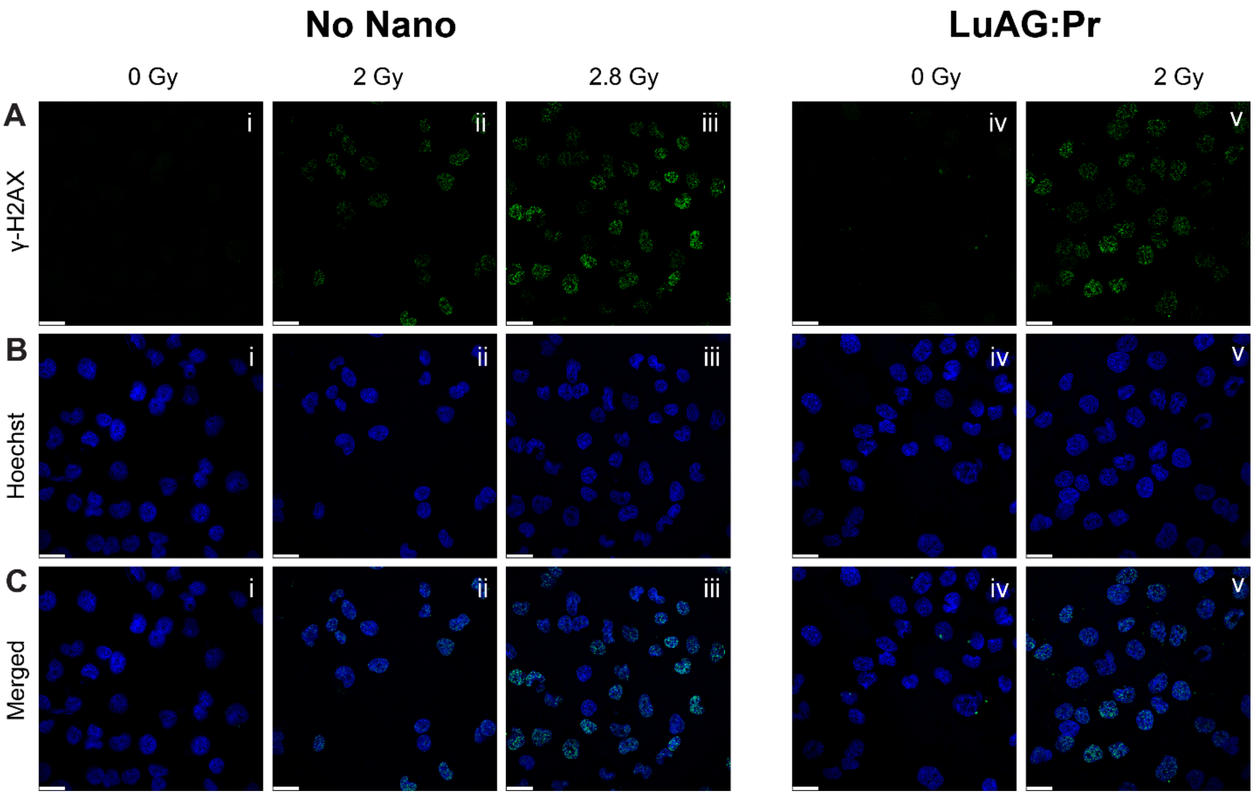

Figure S2: Representative microscopy images showing the **A)** γ-H2AX signal, **B**) Hoechst signal, and **C**) merged images of PANC-1 cells collected 1 hour after X-ray irradiation. In images i-iii, immunofluorescence staining for γ-H2AX foci was performed on PANC-1 cells after they received 0, 2, or 2.8 Gy of X-rays. In images iv-v, cells were first incubated with 0.5 mg/mL $Lu_3Al_5O_{12}@SiO_2$ for 24 hours, then received either 0 or 2 Gy of X-rays. Scale bar = 25 μm.

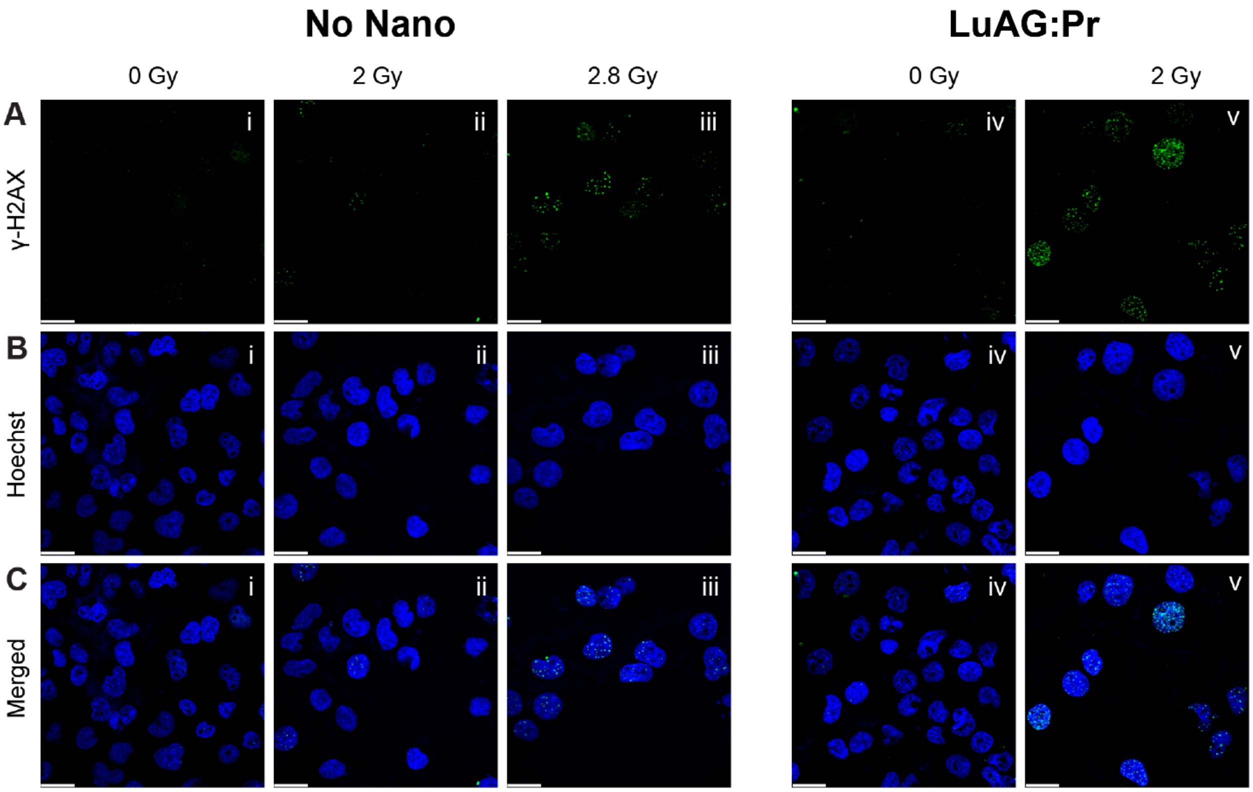

Figure S3: Representative microscopy images showing the **A)** γ-H2AX signal, **B)** Hoechst signal, and **C)** merged images of PANC-1 cells collected 24 hours after X-ray irradiation. In images i-iii, immunofluorescence staining for γ-H2AX foci was performed on PANC-1 cells after they received 0, 2, or 2.8 Gy of X-rays. In images iv-v, cells were first incubated with 0.5 mg/mL $Lu_3Al_5O_{12}@SiO_2$ for 24 hours, then received either 0 or 2 Gy of X-rays. Scale bar = 25 µm

γ-H2AX foci in MIA PaCa-2 cells after X-ray irradiation

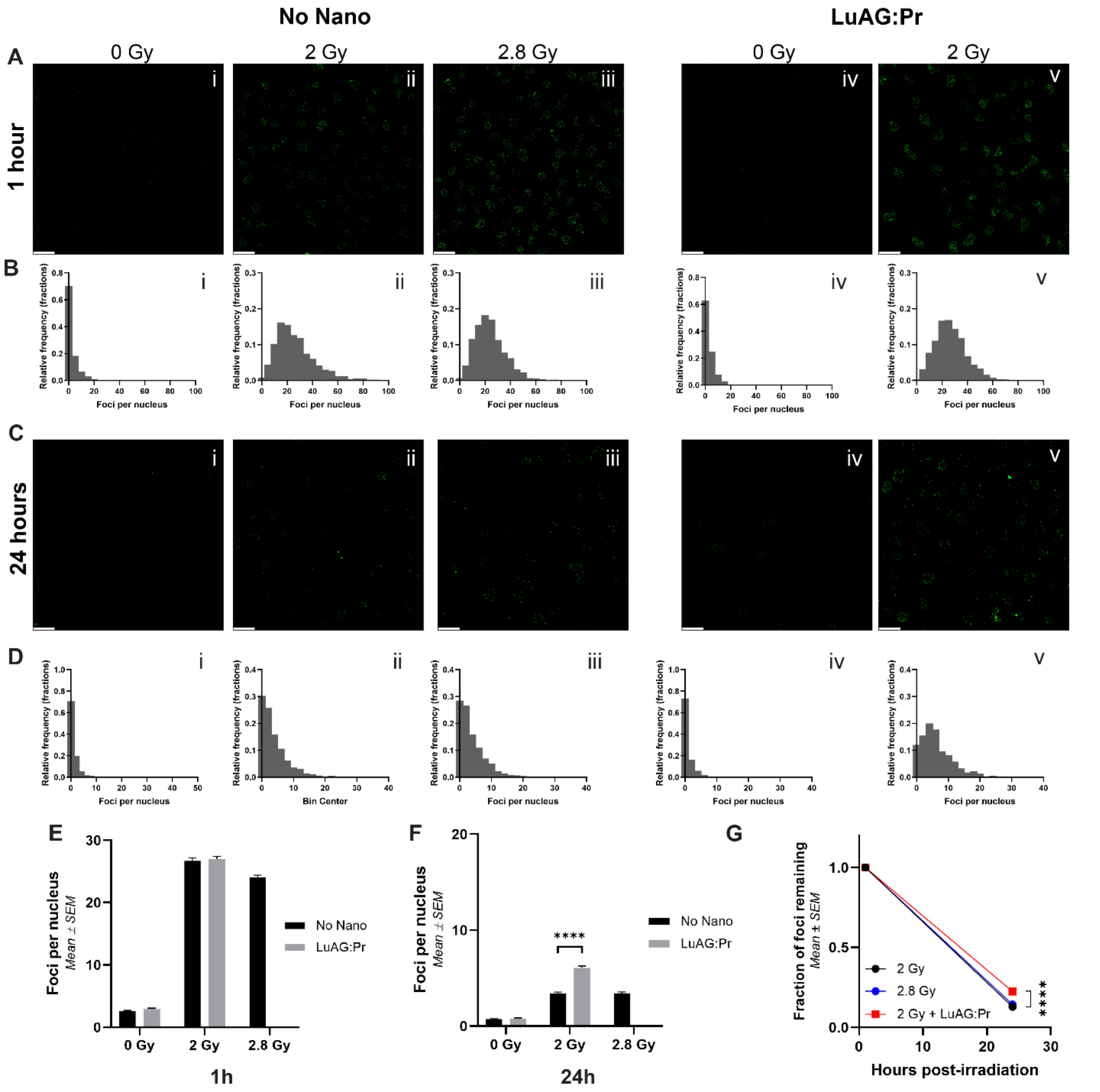

Figure S4: **A)** and **C)** show representative microscopy images taken of MIA PaCa-2 cells collected either 1 hour or 24 hours post-irradiation, respectively. In images i-iii, immunofluorescence staining for γ-H2AX foci was performed on PANC-1 cells after they received 0, 2, or 2.8 Gy of X-rays. In images iv-v, cells were first incubated with 0.5 mg/mL $Lu_3Al_5O_{12}:Pr^{3+}@SiO_2$ for 24 hours, then received either 0 or 2 Gy of X-rays. Scale bar = 25 µm. The number of foci per nucleus was quantified, and the frequency distributions for each condition are shown in panels **B)** and **D)** for cells collected 1 hour and 24 hours after irradiation, respectively. The average number of foci per nucleus (mean ± SEM) in cells irradiated with 2 Gy of X-rays is shown in graphs **E)** and **F)** for samples collected 1 or 24 hours post-irradiation, respectively. **G)** presents the fraction of foci remaining at 24 hours post-irradiation normalized to the number of foci present at 1 hour post-irradiation for the 2 Gy, 2.8 Gy, and 2 Gy + $Lu_3Al_5O_{12}:Pr^{3+}@SiO_2$ conditions. Foci quantification

data was taken from at least 80 nuclei per condition. Statistical significance was assessed using a two-way ANOVA followed by Tukey post hoc test. (*) indicates $p < 0.05$, (**) indicates $p < 0.01$, (***) indicates $p < 0.001$, and (****) indicates $p < 0.0001$.

Efficacy in 3D models of pancreatic cancer under monochromatic synchrotron irradiation

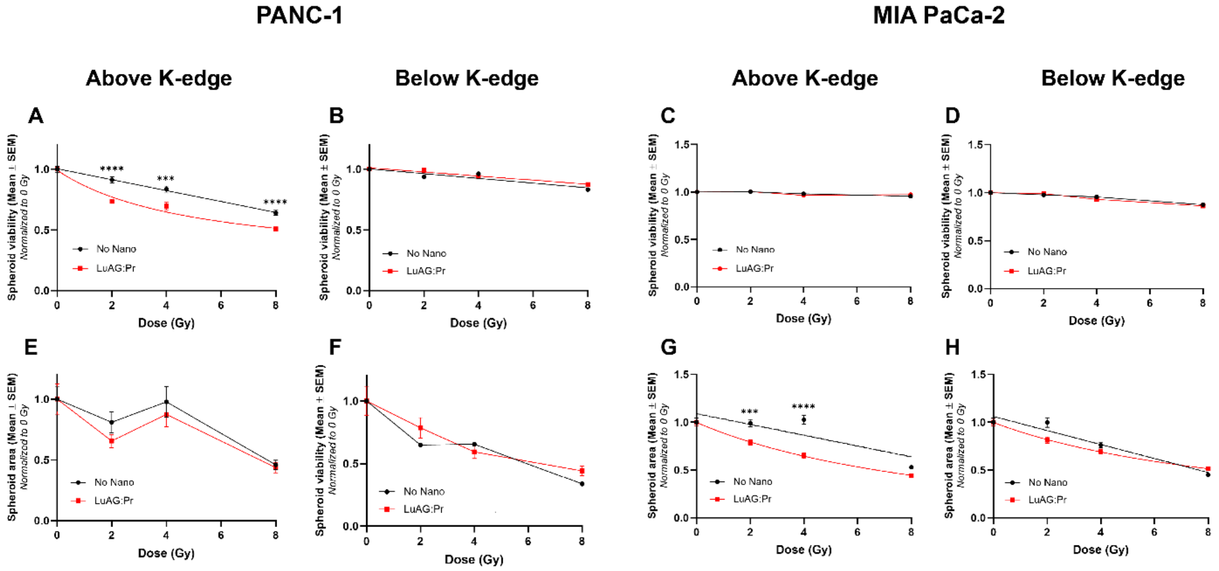

Figure S5: Viability (**A-D**) and area (**E-H**) of PANC-1 (**A-B**, **E-F**) and MIA PaCa-2 (**C-D**, **G-H**) spheroids after irradiation with monochromatic synchrotron radiation of 62.31 keV (**B**, **F**, **D**, **H**) or 64.31 keV (**A**, **E**, **C**, **G**). Data was collected 6 days post-irradiation and is presented as mean ± SEM. The results were normalized to the 0 Gy condition and fitted with a nonlinear regression in Prism according to the [inhibitor] versus response model with three parameters. Statistical significance was assessed using a two-way ANOVA followed by Tukey post hoc test. (*) indicates $p < 0.05$, (**) indicates $p < 0.01$, (***) indicates $p < 0.001$, and (****) indicates $p < 0.0001$.
